# Test on kisspeptin levels in girls with idiopathic central precocious puberty and its significance

**DOI:** 10.1186/1687-9856-2015-S1-P91

**Published:** 2015-04-28

**Authors:** Yu Yang, Xiang-yu Xiong, Li Yang, Li-ing Xie, Hui Huang

**Affiliations:** 1 Department of Endocrinology, The Children's Hospital of Jiangxi Province, Nanchang, China

**Keywords:** Kisspeptin, Idiopathic central precocious puberty, Simple premature thelarche

## Aims

This paper is aimed to explore the significance of plasma kisspeptin level in diagnosis and therapeutic evaluation through the detection of kisspeption level of girls diagnosed with idiopathic central precocious puberty (ICPP) before treatment and after 6-months of treatment and girls with simple premature thelarche (PT).

## Methods

A total of 70 girls including 24 girls diagnosed with ICPP, 21 girls with PT and 25 normal girls were enrolled. ELISA was adopted to detect plasma kisspeptin level.

## Results

The kisspeptin level of ICPP group before treatment (1.80±0.13ng/ml)was higher than those of other groups with significantly statistic difference. The kisspeptin level of ICPP group after 6-months of treatment (1.49±0.21ng/ml) was significantly lower than those before treatment (P<0.05).

## Conclusions

We can conclude that plasma kisspeptin level is related with initiation of pubertal development, and it can be served as important parameter in ICPP diagnosis and therapeutic effect evaluation.

